# Empowering communities for malaria control: Effectiveness of community-led biolarviciding using *Bacillus thuringiensis israelensis* in The Gambia

**DOI:** 10.14202/vetworld.2025.2158-2168

**Published:** 2025-08-02

**Authors:** Babucarr Jassey, Ririh Yudhastuti, Buba Manjang, Ibrahim Touray, Muhammad Rasyid Ridha, Khuliyah Candraning Diyanah, Fitiara Indah Permatasari

**Affiliations:** 1 Department of Public Health, Faculty of Public Health, Universitas Airlangga, Surabaya, Indonesia; 2 Department of Public Health Services, Ministry of Health, The Gambia, West Africa; 3 Department of Public Health, Faculty of Medicine and Health Sciences, Universitas Lambung Mangkurat, Banjarbaru, Indonesia; 4 Department of Nutrition, Faculty of Public Health, Universitas Airlangga, Surabaya, Indonesia

**Keywords:** *Anopheles* mosquitoes, *Bacillus thuringiensis israelensis*, biolarviciding, community participation, larval source management, malaria control, The Gambia

## Abstract

**Background and Aim:**

In The Gambia, malaria transmission persists due to insecticide resistance and residual vector behavior, despite extensive use of indoor residual spraying and insecticide-treated nets. Community-led larval source management using *Bacillus thuringiensis* var. *israelensis* (Bti) offers a sustainable vector control alternative. This study aimed to assess the effectiveness and feasibility of community-led Bti application for reducing *Anopheles* mosquito populations, compared to expert-supervised application and non-intervention control arms.

**Materials and Methods:**

A non-randomized controlled trial was conducted across malaria-endemic regions in The Gambia from 2023 to 2024. Intervention arms included: (1) community-led Bti application, (2) expert-supervised Bti application, and (3) untreated control. Trained volunteers and entomologists applied Bti to breeding sites at weekly or biweekly intervals. Entomological surveys were conducted biweekly to monitor larval, pupal, and adult mosquito densities. Data were analyzed using generalized linear mixed models and negative binomial regression, adjusting for environmental covariates.

**Results:**

By round 10, community-led and expert-supervised interventions achieved 96.8% and 98.6% reductions in larval density, 97.4% and 99.1% reductions in pupal emergence, and 96.2% and 98.8% reductions in adult mosquito populations, respectively. Statistically significant declines in mosquito densities were observed by 2024 (p < 0.001). Community participation enabled high coverage and operational sustainability, with over 85% of participants reporting visible mosquito reduction.

**Conclusions:**

Seasonal Bti application, especially when led by trained community members, significantly suppresses *Anopheles* populations. Although expert-supervised methods yielded slightly higher efficacy, community-led biolarviciding offers a scalable, sustainable, and environmentally safe vector control strategy, supporting The Gambia’s malaria elimination goals.

## INTRODUCTION

Malaria remains a persistent public health chall-enge in The Gambia, largely due to the abundance of seasonal wetlands that serve as optimal breeding grounds for *Anopheles* mosquitoes, the primary vectors of the disease [1–3]. Over the past two decades, signif-icant progress has been made in malaria control through interventions such as indoor residual spraying (IRS) and insecticidetreated nets (ITNs). However, despite these efforts, residual malaria transmission cont-inues, driven by increasing insecticide resistance and behavioral ada-ptations in mosquito populations [4–6].

As *Anopheles* mosquitoes have adapted to bite outdoors and during hours when ITNs are less effective, the limitations of the current adult-targeted control strategies have become more apparent. These developments underscore the need for complementary approaches that target mosquitoes at the larval stage, interrupting the vector lifecycle earlier and enhancing the overall effectiveness of malaria control efforts.

Larval source management (LSM) has emerged as a promising strategy, particularly biolarviciding using *Bacillus thuringiensis* var. *israelensis* (Bti), a microbial larvicide that selectively targets mosquito larvae with minimal environmental impact [7, 8]. Numerous studies across Africa have demonstrated the high efficacy of Bti in reducing *Anopheles* larval populations [9–12]. However, large-scale adoption remains limited, espe-cially in rural and resource-constrained settings, due to the logistical and technical requirements of expert-led implementations.

More recently, attention has turned toward community-led implementation models as a potentially scalable and sustainable alternative. While countries such as Kenya, Rwanda, and Burkina Faso have trialed Bti-based LSM programs, these have mostly relied on centralized or expert-supervised methods. The potential for trained community members to independently carry out effective Bti applications remains underexplored, particularly in West African contexts like The Gambia.

In response to this gap, the present study aimed to evaluate the effectiveness of a seasonal, community-led Bti application strategy for malaria vector control in The Gambia. Using a non-randomized controlled trial conducted over 2 years (2023–2024), we compared larval, pupal, and adult *Anopheles* mosquito densities across three arms: community-led intervention, expert-supervised intervention, and untreated controls.

This is the first study in The Gambia to rigorously assess the performance of community-led biolarviciding using standardized entomological monitoring and statistical modeling. The 2-year longitudinal design, incorporating both unadjusted and adjusted incidence rate ratios (IRRs), provides robust evidence of sustained vector suppression over time. The findings also address critical questions surrounding the feasibility, scalability, and sustainability of decentralized vector control appro-aches in malaria-endemic regions.

Furthermore, this study emphasizes the impo-rtance of community engagement, which has been increasingly recognized as a key determinant of long-term success in public health interventions. Training local volunteers to identify breeding sites and apply Bti not only decentralizes operational responsibilities but also fosters community ownership, increases accept-ance, and improves intervention coverage [8, 13].

Despite the proven efficacy of Bti for mosquito larval control in various African countries, the large-scale implementation of biolarviciding in malaria-endemic, resource-constrained settings like The Gambia remains limited. Most previous initiatives have relied heavily on expert-driven models, which are often cost-prohibitive and logistically complex for sustained national scale-up. Furthermore, while community engagement is widely acknowledged as critical for public health interventions, few studies have rigorously evaluated the feasibility, operational fidelity, and entomological impact of fully community-led Bti application models under real-world field conditions. Particularly in West African contexts, there is a scarcity of empirical data on whether trained local volunteers can independently conduct larval source management with outcomes comparable to expert-led efforts. This gap in implementation research limits the evidence base needed for policy shifts toward decentralized, community-empowered malaria vector control.

This study aimed to evaluate the effectiveness, feasibility, and sustainability of community-led biolarv-iciding using Bti for malaria vector control in The Gambia. Through a 2-year, non-randomized controlled trial, the study compared entomological outcomes, specifically reductions in larval, pupal, and adult *Anopheles* mosquito densities, between three arms: (1) community-led Bti application, (2) expert-supervised Bti application, and (3) an untreated control group. By generating longitudinal entomological data and assessing community participation and acceptability, this study aimed to determine whether community-driven interventions could serve as a viable alternative to expert-led models and inform integrated vector management strategies within national malaria elimin-ation frameworks.

## MATERIALS AND METHODS

### Ethical approval and Informed consent

This study was reviewed and approved by the Gambia Government/MRC Joint Ethics Committee under the Ministry of Health, with approval reference number Et.01/G.10/MoH.01.1/PH.Dir/19.08.021/2023. Written informed consent was obtained from all parti- cipants involved in the study, and all procedures were conducted in accordance with relevant guidelines and regulations.

### Study period and location

The study was conducted from June 2023 to December 2024 in selected malaria-endemic regions of The Gambia, where seasonal wetlands, such as rice fields, floodplains, and stagnant water bodies, serve as primary breeding grounds for *Anopheles* mosquitoes. These environments become especially favorable dur-ing the rainy season, intensifying malaria transmission. Site selection was based on elevated mosquito den-sities, documented malaria incidence, and the logi-stical feasibility of implementing community-based interventions [3]. A detailed overview of the ecological and logistical characteristics of each experimental site is provided in Appendix A.

**Figure 1:**
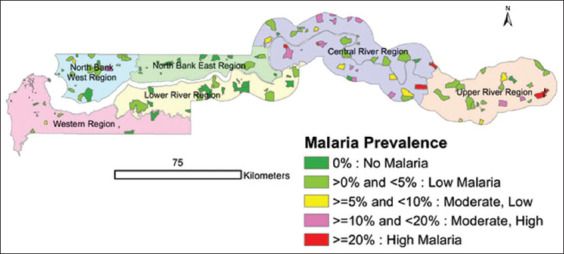
Distribution of *Anopheles gambiae s.l*. reproductive swarms spread across six regions in The Gambia, adapted from the concept of the Malaria Indicator Survey. The figure illustrates the distribution of *Anopheles gambiae s.l*. reproductive swarms across six administrative regions of The Gambia: the North Bank West Region, the North Bank East Region, the Western Region, the Lower River Region, the Central River Region, and the Upper River Region. The study was conducted across selected intervention and control villages in these regions. On the map, red dots represent intervention villages where *Bacillus thuringiensis* var. *israelensis* applications were implemented, while blue dots indicate control villages where no intervention was applied. The intervention and control communities were approximately 23 km apart, as shown in a satellite image captured using Google Earth Pro 7.3.4.8642. The green circles denote sites with confirmed *Anopheles gambiae s.l*. reproductive swarms (positive swarming locations), whereas the red circles indicate locations where no swarming activity was detected (negative swarming locations).

The Gambian climate features distinct dry and wet seasons, with peak malaria transmission occurring during and after the rainy period. The study sites encompassed both rural and semi-urban communities where malaria prevention largely relies on insecticide-based methods such as ITNs and IRS. However, persi-stent malaria transmission in these areas indicates a need for complementary strategies like biolarviciding. To support implementation, village leaders, public hea-lth officials, and community members were engaged early in the planning process. The spatial distribution of intervention and control communities, along with confirmed Anopheles swarm locations across the six study regions, is illustrated in Figure 1.

### Design of intervention arms

A non-randomized controlled trial was employed, consisting of three intervention arms:

1. Community-led Bti application
2. Expert-supervised Bti application
3. Control group with no intervention.

Each arm was assigned to a distinct mosquito breeding site to prevent overlap. Baseline assessments were used to identify and map larval habitats, which included stagnant pools, irrigation ditches, and natural depressions that retain water during the rainy season.

### Site mapping and application frequency

Baseline surveys and habitat mapping were conducted jointly by the entomology team and local health workers. Breeding sites were selected based on the presence of *Anopheles* larvae, the persistence of water during the wet season, and accessibility for routine application. High-risk areas such as rice fields and irrigation canals were also identified using satellite imagery and local input.

Application frequency was tailored to local enviro-nmental conditions. In areas like the Lower River Region and North Bank East, where water bodies were more persistent, weekly applications were necessary. In contrast, biweekly applications were sufficient in regions like the Western and Upper River Regions due to faster drying of habitats or logistical constraints. This strategy ensured effective larval suppression while optimizing resource use.

### Roles and responsibilities in each arm

In the expert-supervised arm, trained entomo-logists and field technicians applied Bti following stand-ardized protocols. The community-led intervention relied on trained local volunteers, – such as rice farmers and village health workers, – who were familiar with the ecological conditions and breeding site locations. The control group received no treatment, serving as a baseline for the natural dynamics of mosquitoes. Adequate spatial separation was maintained between arms to avoid cross-contamination.

### Selection of Bti applicators

Bti applicators were chosen through a collabora-tive process involving the research team, local health authorities, and community leaders. Participants were selected based on prior experience in health initiatives, such as work with malaria control programs or village health services. Rice farmers were specifically included for their knowledge of water systems and personal stake in vector reduction. Selection criteria emphasized commitment, local residence, and reliability.

Professional entomologists led the expert-supe-rvised arm and provided quality oversight for the community-led arm. Both groups were required to maintain detailed records, documenting Bti quan-tities used, sites treated, and any operational issues encountered.

The integration of trusted community figures, such as farmers and health workers, was central to the program’s success. Their familiarity with local geography and rapport with residents helped ensure effective coverage and community buy-in.

### Operational structure of the intervention

A structured coordination framework was put in place to manage activities and ensure adherence. The expert-supervised arm followed a centralized model with oversight from the research team, while the community-led arm employed decentralized management with zone-based assignments and local supervisors.

Community-led teams reported progress weekly, and expert-supervised teams submitted daily updates for more intensive tracking. Regular refresher sessions were conducted in both arms to reinforce correct storage, handling, and application of Bti.

### Bti as larvicide

The larvicide used was Bti, a microbe-derived formulation specific to mosquito larvae with minimal environmental impact. A water-dispersible granule formulation containing 3000 International Toxic Units/mg was selected for its stability, ease of use, and safety for non-target species.

A dose of 0.3 kg/ha was applied, in line with manufacturer recommendations. Bti was mixed with water and sprayed using backpack sprayers to ensure even distribution. Applications were conducted weekly based on the mosquito life cycle to maintain larval suppression. Applicators were trained to calibrate equipment for consistent flow rates and coverage.

### Training and equipment calibration

All applicators participated in a comprehensive 3-day training course covering both theory and practice.

- Day 1: Malaria transmission, mosquito biology, and principles of LSM
- Day 2: Breeding site identification, Bti safety, dosage calculations, and handling
- Day 3: Field exercises, including mapping, calibr-ation, and supervised spraying.

Participants were evaluated and given logbooks and reference materials. Refresher trainings were held biweekly. Trainers, including entomologists and public health experts, ensured that trainees could distinguish between mosquito stages and identify target habitats.

Calibration was emphasized as essential for accu-rate application of larvicides. Participants learned to adjust nozzle settings, measure correct Bti-to-water ratios and maintain consistent spray speed. A trial run was conducted to practice techniques and troubleshoot potential issues, ensuring proper application across all sites.

### Entomological surveillance

Biweekly entomological surveys were conducted to assess the impact of the intervention on mosquito populations.

- Larval Monitoring: Conducted using standard dipping methods (10 dips/site), followed by species-level identification of collected larvae
- Adult Monitoring: Carried out using the Centers for Disease Control (CDC) light traps placed near sleeping areas in households adjacent to intervention zones [14, 15].

Captured mosquitoes were sorted and counted to assess temporal changes in adult mosquito density. These surveillance activities were key to determining the effectiveness of Bti applications across treatment arms.

### Statistical analysis

Data from entomological surveys were analyzed using Generalized Linear Mixed Models to measure changes in larval, pupal, and adult mosquito counts over time. The effect of Bti treatment was compared across intervention and control arms, with adjustments for climatic variables such as rainfall and temperature.

Spatial maps and graphical representations were utilized to visualize the dynamics of mosquito popu-lations. Comparative analysis between the community-led and expert-supervised arms further clarified the relative efficacy of each approach.

### Covariates and analytical framework

Key covariates included:

- Survey round
- Temperature (°C)
- Rainfall (mm)
- Proximity to water sources (km)
- Intervention arm (control, expert-supervised, community-led).

These variables were selected due to their known influence on mosquito population dynamics [refs]. Environmental data were sourced from nearby meteorological stations. Proximity to breeding sites was included to reflect ecological risk. Random effects accounted for clustering at the household and village levels, enabling precise estimates of Bti treatment impact while controlling for heterogeneity.

## RESULTS

### Impact of Bti on larval and pupal stages of *Anopheles* mosquitoes

The application of Bti led to a significant reduction in *Anopheles* larval and pupal densities in intervention areas compared to the control. Pre-intervention asse-ssments revealed widespread breeding habitats across all study sites. However, after a few rounds of Bti appl-ication, larval densities decreased markedly in both the expert-supervised and community-led arms. By the fifth application round, larval densities had dropped by over 70% in treated areas, while control sites continued to show persistently high levels.

**Figure 2:**
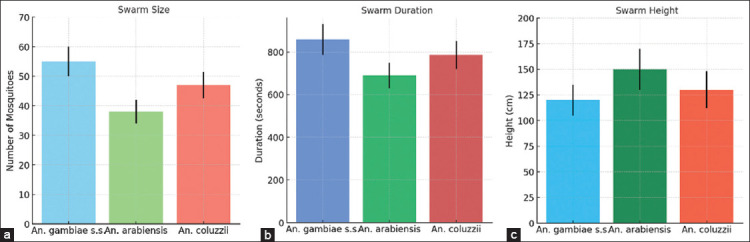
Characteristics of *Anopheles gambiae s.l*. reproductive swarms by species. (a) Size, (b) Duration, and (c) Height.

As shown in Table 1, both intervention arms exhibited a steady decline in larval and pupal densities across 10 application rounds, whereas control areas remained largely unchanged throughout the study. The characteristics of *Anopheles gambiae* *s.l*. reproductive swarms, including swarm size, duration, and height, are also summarized in Figure 2.

**Table 1:** Mean *Anopheles* larval and pupal densities (per dip) across the study arms.

Round	Community- led Bti	Expert- supervised Bti	Control (No Bti)
Baseline	15.4 ± 2.1	14.9 ± 2.3	15.7 ± 2.4
Round 1	10.6 ± 1.8	9.5 ± 1.7	14.8 ± 2.1
Round 3	5.2 ± 1.4	4.1 ± 1.2	14.2 ± 2.0
Round 5	2.8 ± 0.9	1.7 ± 0.6	13.5 ± 1.9
Round 7	1.3 ± 0.4	0.8 ± 0.3	12.8 ± 1.7
Round 10	0.5 ± 0.2	0.2 ± 0.1	12.5 ± 1.5

The data in Table 1 clearly demonstrate a consistent decrease in Anopheles larval densities in both intervention arms, with the expert-supervised group achieving the most significant reductions. By round 10, larval concentrations were nearly eliminated in treated areas, whereas the control maintained relatively high numbers. A parallel reduction in pupal densities was observed, with pupal emergence nearly abolished from round 5 onward in Bti-treated locations . Bti=*Bacillus thuringiensis* var. *israelensis*

### Impact on adult *Anopheles* mosquito populations

The decline in immature mosquito stages corres-ponded with a notable reduction in adult mosq-uito densities. CDC light traps placed near households demo-nstrated sharp declines in adult *Anopheles* mosquitoes in areas where Bti was applied, while control areas maintained consistently high adult mosquito counts.

Table 2 summarizes the mean number of adult mosquitoes captured per household per night, showing substantial decreases in both treatment arms. By the tenth round, the average adult mosquito count per household had dropped by more than 95% in intervention areas, whereas no significant changes were seen in the control group. These findings underscore Bti’s role in effectively interrupting the mosquito life cycle and reducing adult vector burden.

**Table 2:** Mean number of adult *Anopheles* mosquitoes captured per household at night.

Round	Community- led Bti	Expert- supervised Bti	Control (No Bti)
Baseline	42.6 ± 4.8	41.3 ± 4.5	43.2 ± 4.9
Round 1	30.5 ± 3.2	28.7 ± 3.1	42.1 ± 4.7
Round 3	18.4 ± 2.6	14.9 ± 2.3	41.2 ± 4.5
Round 5	9.3 ± 1.7	5.6 ± 1.2	40.7 ± 4.4
Round 7	4.7 ± 0.9	2.1 ± 0.6	39.8 ± 4.2
Round 10	1.2 ± 0.3	0.5 ± 0.2	38.9 ± 4.0

Bti=*Bacillus thuringiensis* var. *israelensis*

### Comparison of community-led and expert-supervised Bti applications

Both community-led and expert-supervised Bti interventions achieved significant reductions in mos-quito populations. However, expert-led applications were marginally more effective. By the tenth round, the exp-ert-supervised group had achieved a 98.8% reduction in adult mosquito density (p < 0.001), compared to a 96.2% reduction in the community-led group (p < 0.001).

The difference in performance likely resulted from more consistent application techniques and dos-ing accuracy by expert teams. Community-led effo-rts, though slightly less precise, still demonstrated impressive efficacy. Table 3 provides a side-by-side comparison of reduction rates for larval, pupal, and adult mosquito stages across the three study arms, reinforcing the strong potential of both strategies.

**Table 3:** Comparative effectiveness of community-led and expert-supervised Bti application

Indicator	Community- led Bti	Expert- supervised Bti	Control (No Bti)
Larval density reduction (%)	96.8	98.6	20.5
Pupal emergence reduction (%)	97.4	99.1	18.2
Adult mosquito reduction (%)	96.2	98.8	10.0

Bti=*Bacillus thuringiensis* var. *israelensis*

### Year-wise effectiveness and statistical analysis

Further analysis of *Anopheles gambiae s.l*. densities across 2023 and 2024 using unadjusted and adjusted negative binomial regression models is pre-sented in Table 4. In 2023, statistical significance was observed only in the adjusted model (p = 0.018), with no significant effect noted in the unadjusted analysis (p = 0.311). This suggests a modest early impact, possibly due to environmental variability, initial implementation gaps, or lag effects in population growth.

**Table 4:** *Anopheles gambiae s.l*. density versus Bti application per home in 2023 and 2024, as determined by unadjusted and adjusted negative binomial regression analysis, comparing the control and target arms.

Factors		Adjusted analysis		Unadjusted analysis	
		
Years	Intervention	IRR (95% CI)	p-value	IRR (95% CI)	p-value
2023	Target	Ref	0.018	Ref	0.311
	Control	0.84 (0.28–1.31)		1.41 (0.72–1.52)	
2024	Target	Ref	0.001	Ref	<0.001
	Control	0.72 (0.33–1.19)		0.65 (0.49–0.88)	

Bti=*Bacillus thuringiensis* var. *israelensis*, CI=Confidence interval, IRR=Incidence rate ratio, Ref=Reference

In contrast, 2024 results showed statistically significant reductions in both adjusted and unadjusted models (p < 0.001), supporting the cumulative and sustained impact of repeated Bti applications. The progressively declining IRRs confirm the long-term effe-ctiveness of community-led and expert-supervised Bti strategies in suppressing malaria vector populations.

### Community engagement and sustainability

Community participation proved essential to the success of the intervention. Local volunteers played an active role in identifying breeding sites, applying Bti, and monitoring mosquito activity. Post-intervention feed-back showed that over 85% of participants perceived a visible reduction in mosquito nuisance, and 78% expre-ssed confidence in Bti’s long-term benefits.

Despite these achievements, some operational challenges were reported, such as difficulties accessing remote breeding habitats and occasional delays in Bti supply. These limitations highlight the need for enh-anced logistical planning, more frequent refresher training, and stronger community ownership to ensure the sustainability of decentralized Bti interventions.

### Visual summary of intervention outcomes

**Figure 3:**
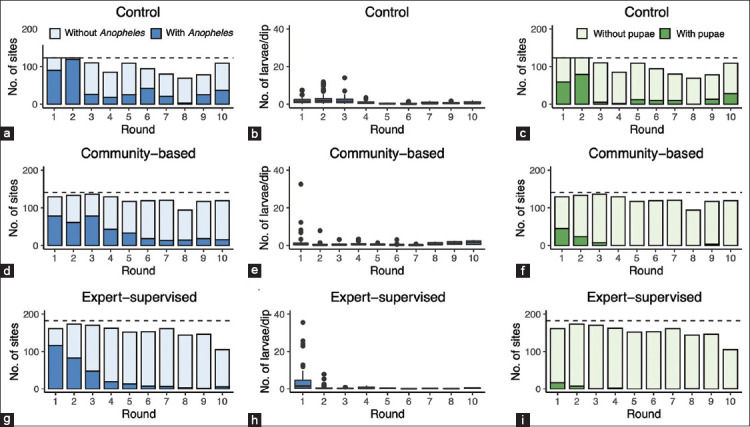
Impact of *Bacillus thuringiensis* var. *israelensis* (Bti) on adult *Anopheles* mosquito populations in different study arms: Panels (a–c) represent the control group, (d–f) depict the community-led Bti application, and (g–i) illustrate the expert-supervised Bti intervention. The images show the progressive reduction in adult *Anopheles* mosquito densities across the study period, with the highest suppression observed in the expert-supervised intervention.

Figure 3 visually demonstrates the effect of Bti on adult mosquito populations. Panels a–c (control group) show persistently high mosquito densities, indicating no natural reduction. Panels d–f (community-led arm) dep*ict a marked decrease in mosquito numbers, validating the capability of trained local volunteers. Panels g–i (expert-supervised arm) show the sharpest decline, reflecting precision and dosing consistency.

**Figure 4:**
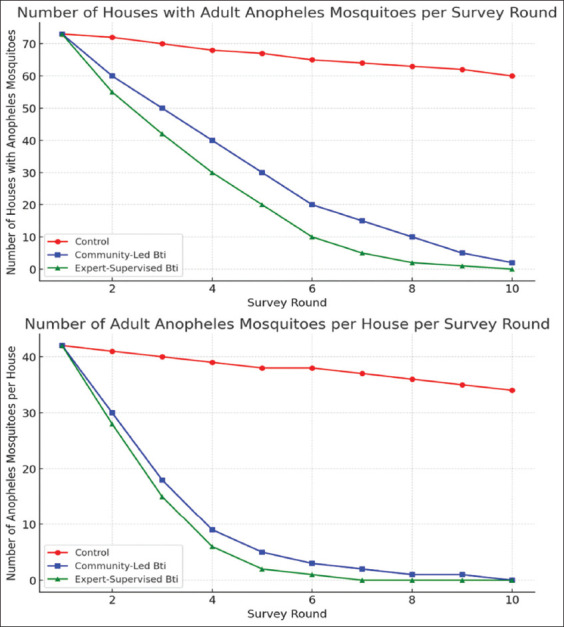
(a) Number of houses with adult *Anopheles* mosquitoes detected during each biweekly survey round- comparing control, community-led Bti, and expert-supervised Bti interventions. (b) Mean number of adult *Anopheles* mosquitoes per house per house round-demonstrating the decline in mosquito populations across the three study arms. Comparisons are shown for control, community-led, and expert-supervised Bti interventions. Data show consistent reductions in both treated arms compared with controls. Bti=*Bacillus thuringiensis* var. *israelensis*.

Figure 4 further reinforces these findings. Panel “a” shows the number of houses with adult mosquitoes over 10 rounds – remaining high in the control arm but steadily declining in both intervention arms. Panel b displays the mean number of adult mosquitoes per household, with the expert-supervised group achieving near-complete elimination and the community-led group demonstrating substantial, sustained reductions.

Overall, the results confirm that Bti application, – whether expert-led or community-driven, – significantly reduces *Anopheles* larval and adult populations. While expert-supervised application provided slightly superior results, the community-led approach demonstrated high feasibility, strong community acceptance, and scalability. These findings position community-led biolarviciding as a sustainable, impactful strategy for malaria vector control in The Gambia and similar ende-mic regions.

## DISCUSSION

### Effectiveness of Bti-based vector control

This study evaluated the effectiveness of both community-led and expert-supervised applications of Bti for malaria vector control in The Gambia. Results showed significant reductions in larval, pupal, and adult *Anopheles* mosquito populations in treated areas compared to control sites. These findings underscore the potency of Bti as a biological larvicide and support its inclusion in integrated malaria vector control stra-tegies. Notably, the study demonstrated that trained community members can effectively administer Bti with considerable success. Although expert-supervised interventions achieved slightly greater reductions, the strong performance of community-led efforts highlights their practicality, scalability, and potential for long-term sustainability.

### Alignment with existing literature

The outcomes of this study are consistent with findings from other malaria-endemic regions. Previous research by Hakizimana *et al*. [8], Mutero *et al*. [10], Mboera *et al*. [14], Gonçalves *et al*. [16], Shanks *et al*. [17], and Rurangirwa *et al*. [18] in Tanzania, Kenya, and Rwanda has confirmed the capacity of Bti to suppress larval and pupal mosquito stages, leading to declines in adult populations. The near-elimination of pupae by the fifth round of Bti application in this study further affirms its ability to disrupt the mosquito life cycle when applied consistently. Importantly, unlike prior studies that primarily relied on expert-led implementation, this research provides strong evidence that communities can independently manage Bti interventions with mini-mal supervision [8]. This highlights a viable pathway for empowering communities in the ongoing fight against malaria.

### Comparative performance and operational challenges

While both approaches were effective, the expert-supervised arm achieved marginally higher reductions in mosquito densities. This is likely due to more consistent adherence to application protocols and accurate dosage delivery. In contrast, the community-led strategy faced some logistical limitations, including occasional del-ays in application and uneven distribution of Bti [19]. These challenges suggest that, although community-led programs are viable, their impact could be further optimized through ongoing training, technical oversight, and structured quality assurance [20, 21].

### Lessons from other community-based programs

Several African countries have reported succe-sses with community-based Bti programs [8, 10, 14]. However, other studies have highlighted common obstacles such as inconsistent application coverage, variable volunteer engagement, and supply chain con-straints. For instance, experiences in rural Ethiopia and western Kenya revealed that volunteer fatigue and inconsistent training undermined uniformity in Bti application [10, 19]. Similar implementation barriers were observed in this study, further reinforcing the importance of refresher training, local supervision, and reliable logistical support to maintain program fidelity and impact.

### Environmental and strategic advantages of Bti

One of Bti’s core advantages is its environmental safety and high specificity to mosquito larvae, minimizing unintended effects on non-target species [12, 22]. Unlike conventional insecticides, Bti does not promote resistance development and has minimal ecological disruption. This makes it an ideal supplement to standard interventions, such as ITNs and IRS [13, 23]. Given rising concerns about insecticide resistance in *Anopheles* populations, incorporating Bti into national vector control programs presents a sustainable and ecologically sound alternative [3]. Furthermore, Bti’s biodegradability makes it suitable for use in sens-itive ecosystems, including rice paddies and wetland habitats [24].

### Community involvement and program sustainability

Community participation was a cornerstone of the intervention’s success. Local volunteers played key roles in identifying breeding sites, applying Bti, and monitoring outcomes. Post-intervention surveys revealed widespread community support: over 85% of participants noticed a reduction in mosquito nuisance, and 78% believed Bti was effective for long-term malaria control. Sustaining this level of engagement will require continued capacity building, adequate reso-urce allocation, and support from local governance structures [20, 25, 26]. Enhancing ownership through participatory planning and incentive mechanisms could strengthen the long-term viability of community-led biolarviciding programs.

### Implications for future implementation

The findings of this study demonstrate that community-led biolarviciding is both feasible and effective in malaria-endemic settings such as The Gambia. To maximize impact, future efforts should include cost-effectiveness studies, long-term monit-oring, and an exploration of how Bti can be combined with other vector control tools, including habitat modification and larval source reduction. The results strongly support seasonal Bti application, paired with community mobilization, as a practical and sustainable strategy for malaria control.

This research provides robust field-based evidence that both expert-led and community-driven Bti applications can significantly suppress *Anopheles* mosquito populations. The community-led model, in particular, shows strong potential for expansion in low-resource settings due to its cost-effectiveness and local acceptability. However, implementation success depends on adaptive management strategies that address training, technical oversight, and regional varia-bility. These findings advocate for the incorporation of biolarviciding into national malaria strategies as a complementary intervention, especially in the face of escalating insecticide resistance. Further research should prioritize randomized trials, economic evaluations, and policy frameworks that support decentralized vector control initiatives in endemic regions.

### Policy implications

This study presents a practical framework for incorporating community-led Bti distribution into existing public health structures such as village health committees and district health teams. With modest investments in supply chains, training, and supervision, the approach could be expanded under The Gambia’s national malaria strategy. Furthermore, aligning Bti inte-rventions with existing environmental or agricultural programs could improve cost-efficiency and foster community ownership.

### Study limitations

Several limitations should be considered when interpreting the findings. The non-randomized design may have introduced selection bias, as intervention allocation was based on community readiness and logistical considerations rather than random assign-ment. This could have led to baseline differences in mosquito density or environmental characteristics. Although statistical adjustments were made for rain-fall, temperature, and proximity to breeding sites, unmeasured confounders such as vegetation cover, human activity patterns, or microclimatic differences may still have influenced the results. Additionally, varia-bility in ecological conditions may have affected the uniformity of Bti performance across regions, especially in areas with ephemeral breeding sites. To enhance the strength of future evidence, randomized controlled trials with extended follow-up and cost-benefit analysis are recommended.

## CONCLUSION

This study provides compelling evidence that seasonal application of Bti, whether community-led or expert-supervised, is highly effective in suppressing *Anopheles* mosquito populations in malaria-endemic regions of The Gambia. Over 10 application rounds, larval and pupal densities in treated areas were reduced by more than 96%, with adult mosquito populations declining by up to 98.8% in the expert-supervised arm and 96.2% in the community-led arm. The near-elimination of pupal emergence by the fifth round highlights Bti’s efficacy in interrupting mosquito development cycles.

A key strength of this study lies in its 2-year longitudinal design, use of standardized entomological monitoring, and the inclusion of both adjusted and unadjusted statistical models. The direct comparison between community-led and expert-supervised interv-entions under real-world conditions also strengthens the generalizability of the findings. The involvement of trained local volunteers demonstrated that dece-ntralized, community-driven biolarviciding is not only feasible but also operationally effective and socially accepted.

These results support the integration of Bti into The Gambia’s national malaria control strategy as a complementary and environmentally sustainable inte-rvention, particularly in light of growing insecticide resistance. Empowering communities through capacity building, logistical support, and participatory implem-entation models may enhance long-term vector control and accelerate progress toward malaria elimination.

Future research should explore cost-effectiveness analyses, optimize training protocols, and evaluate inte-gration with other LSM strategies to further strengthen the scalability and sustainability of this approach.

## DATA AVAILABILITY

All data generated and analyzed during this study are fully presented within the article. Any supp-lementary materials, including raw datasets, field protocols, or additional documentation, can be made available by the corresponding author upon reasonable request. Access will be granted for academic and non-commercial purposes in accordance with ethical guidelines and data-sharing policies.

## AUTHORS’ CONTRIBUTIONS

BJ: Conceived and designed the study, led the implementation, performed data interpretation, and drafted the manuscript. RY: Supervised the research process, provided critical revisions, and guided overall study design and analysis. BM: Coordinated field implementation in The Gambia, ensured alignment with national malaria control activities, and facilitated stakeholder engagement. IT: Conceptualized the study alongside BJ, managed daily field operations, includ-ing team coordination, data collection, facilitated stake-holder engagement, and site logistics. MRR: Contributed to methodological refinement, advanced statistical analysis, manuscript revision, and critical review of entomological data. KCD: Data sorting, preliminary analysis, and preparation of visual data presentations. FIP: Data management, entry accuracy, coding, and supported data quality assurance. All authors have read and approved the final manuscript.

## Appendix

**Table A1:** Characteristics of the experimental sites in The Gambia.

Characteristic	NBWR	NBER	WR	LRR	CRR	URR
Dominant land use	Rice fields and wetlands	Floodplains, swamps	Urban, peri-urban	Irrigated farmlands	Seasonal wetlands	Riverine and rice fields
Malaria endemicity	Moderate	High	Moderate	High	Very High	Very High
Average rainfall (mm/year)	900–1,200	1,000–1,300	1,100–1,400	1,000–1,200	900–1,100	800–1,000
Primary mosquito breeding sites	Marshlands, floodplains	Irrigated fields and ponds	Urban drains and wetlands	Rice paddies and swamps	Flooded plains and streams	Riverbanks and irrigation channels
Intervention type	Community-led Bti	Expert-supervised Bti	Community-led Bti	Community-led Bti	Expert-supervised Bti	Community-led Bti
Distance to nearest health facility (km)	5–15	10–20	2–10	8–18	12–25	15–30
Main economic activities	Farming, fishing	Agriculture, livestock	Trade, services	Farming, fishing	Farming, animal husbandry	Farming, fishing
Bti application frequency	Weekly	Weekly	Biweekly	Weekly	Weekly	Biweekly
Bti sprayer team composition	Community volunteers	Trained entomologists	Community volunteers	Community volunteers	Trained entomologists	Community volunteers
Monitoring method	Larval and adult surveys	Larval and adult surveys	Larval and adult surveys	Larval and adult surveys	Larval and adult surveys	Larval and adult surveys
Reporting schedule	Weekly	Daily	Weekly	Weekly	Daily	Weekly

This table provides an overview of the geographical, ecological, and socioeconomic characteristics of the study sites where Bti interventions were applied. The Central River Region (CRR) and Upper River Region (URR) exhibited the highest malaria endemicity due to extensive flooded plains and riverine habitats, making them ideal locations for expert-supervised Bti applications. Conversely, community-led interventions were implemented in regions with moderate-to-high malaria prevalence, such as the North Bank West Region (NBWR), Lower River Region (LRR), and Western Region (WR), to assess the feasibility of decentralized malaria control efforts. The diversity of breeding habitats across the sites highlights the need for adaptive larval source management strategies, as urban drainage systems in WR require different application approaches compared to seasonal wetlands in CRR and URR. Understanding these characteristics is essential for optimizing Bti application and ensuring long-term success in malaria vector control programs. Bti=*Bacillus thuringiensis* var. *israelensis*
